# Clinical evaluation of patients living with obesity

**DOI:** 10.1007/s11739-023-03263-2

**Published:** 2023-04-29

**Authors:** Laurence J. Dobbie, Claudia Coelho, James Crane, Barbara McGowan

**Affiliations:** Guy’s and St Thomas’ NHS Foundation Trust, London, UK

**Keywords:** Obesity, Overweight, Clinical evaluation

## Abstract

Obesity is a significant public health concern which is implicated in cardiometabolic disease, mechanical complications and psychiatric sequelae. BMI is currently used for diagnosis; however, it has limited sensitivity for adiposity in certain circumstances. This has led to the development of risk stratification tools like the Edmonton Staging criteria and the Kings Obesity Staging Criteria: these facilitate and guide comprehensive obesity-related complication assessment. Healthcare professionals working within obesity clinics should adopt evidence-based communication strategies, including shared decision-making, motivational interviewing, and realistic goal setting. It is also vital to avoid weight-stigmatising terminology in all aspects of care, as this can negatively impact patients. Primary care plays an essential part in obesity care and should work to promptly identify cases, initiate treatment and forward on to specialist services where appropriate. Clinical evaluation of the patient living with obesity should take a holistic approach and involve input from bariatric physicians, dietitians, psychologists, and bariatric surgeons, wider members of the multi-disciplinary team should be involved where needed. Clinicians should take a detailed history, examination and order laboratory tests to investigate for complications. Overall, with appropriate evaluation, these assessments can guide patient management and facilitate long-term improvement in health.

## Introduction

Obesity is a major public health concern, with prevalence increasing globally. Obesity is a disease in itself; it is an established risk factor for multiple different conditions including cardiovascular disease (CVD) (i.e., coronary heart disease, stroke, hypertension), metabolic disease (i.e., type 2 diabetes (T2DM), non-alcoholic fatty liver disease (NAFLD)), osteoarthritis, cancer and obstructive sleep apnoea syndrome (OSA) [1]. Global data indicate that ~ 1.9 billion adults are living with overweight and ~ 650 million adults are living with obesity [2]. As reported by the world health organisation (WHO), obesity and overweight are the 5.^th^ leading cause of mortality, with 2.8 million adults dying annually due to obesity and its related complications [3]. It is, therefore, crucial to have robust strategies in place for the clinical assessment of patients living with obesity. This is particularly important given novel emerging therapies (semaglutide, tizepatide) and newer surgical approaches for the management [4]. This review will provide an overview of the clinical evaluation of the patient with obesity, providing insight into adiposity, obesity staging, disease risk, healthcare professionals approach in managing weight and an overview of the assessment [5].

### Obesity diagnosis and determination of disease risk

The Term BMI, or body mass index, was coined by Ancel Keys in 1972 based on research by the Belgian statistician Adolphe Quetelet. BMI (kg/m2) is a crude measure of body composition and is calculated by dividing a person’s weight (kg) by the square of their height (metres) and had become established as the primary clinical tool for defining obesity [6]. BMI can be helpful to categorise different levels of obesity in adults (Table 1) and is associated with various health outcomes. For every 5 kg/m2 increase in BMI mortality rate increases by 30%; a BMI of 40–45 corresponds to a ~ 10-year reduction in life expectancy, whereas BMI of 20–24 has the lowest associated mortality [7, 8]. In addition, BMI is an established risk factor for diabetes, cancer NAFLD, polycystic ovary syndrome (PCOS), CVD, OSA, psychological conditions and mechanical complications (i.e., osteoarthritis) [9, 10]. However, BMI is only a rough correlate of true obesity (properly defined as an excess of adipose tissue) limited by its inability to differentiate fat and lean mass. BMI cannot account for ethnicity, sex, and age-related difference in adiposity or for variation in muscle mass. It offers no insight into adipose differentiation (relative volumes of pathogenic ectopic and visceral fat compared to more benign subcutaneous fat) nor extend of obesity-related disease. BMI has high specificity (0.90) but low sensitivity (0.50) for adiposity; the measure misclassifies 50% of individuals with excess adiposity as having normal adiposity [11, 12]. In addition, at lower BMI levels, the correlation between BMI and densitometry determined body fat % is low hindering clinical utility [13, 14]. Overall, additional tools are needed to risk stratify patients in these cases where BMI is known to be sub-optimal [12].

**Table 1:** WHO classification of weight status

Classification		BMI cut-off (Caucasians)	BMI cut-off (Asian ethnicity)
Underweight		< 18.5	< 18.5
Normal Weight		18.5–24	18.5–22.9
Overweight		25.0–29.9	23–24.9
Obese	Obese Class I	30.0–34.9	25–29.9
	Obese Class II	35.0–39.9	
	Obese Class III	≥40.0	

Adapted from: https://www.ncbi.nlm.nih.gov/books/NBK513285/figure/article-35323.image.f1/ [77]

#### Central obesity

Central obesity measurement can provide further insight into obesity-related risk determination. This is important as central obesity acts as a surrogate for pathogenic visceral and ectopic fat depots. For instance, waist–hip ratio (WHR) independently predicts CVD mortality, and individuals with raised waist circumference (WC) are more likely to develop cardiometabolic complications (T2DM, dyslipidaemia, metabolic syndrome, hypertension) [15, 16]. BMI and WHR also independently predict insulin resistance, indicating the utility of the concomitant assessment of both BMI and central obesity [17]. However, as delineated by the Emerging Risk Factors Collaboration epidemiological research (n = 221,934), BMI, WC and WHR do not improve CVD prediction when data on systolic blood pressure, diabetes history and lipid profile are available. Therefore, the optimal pathway is conducting a comprehensive clinical evaluation [18].

#### Staging criteria

As discussed, BMI has shortcomings, with individuals with obesity defined by BMI sometimes having lower complication risk than those with overweight or normal weight. This is because adiposity levels at a given BMI can vary between individuals, making BMI a poor indicator. Clinical staging criteria have been devised to determine obesity-related complication risk and are an essential component of clinical evaluation. Criteria include the Edmonton obesity staging criteria, cardiometabolic staging system and Kings obesity staging criteria [19]. These criteria are required to prioritise patients and streamline them to appropriate therapeutic pathways, i.e., lifestyle, pharmacotherapy or bariatric surgery [20].

The Edmonton obesity staging system (EOSS) is an obesity staging system (Table 2) accounting for obesity-related risk factors (i.e., Glucose, cholesterol, Triglycerides, HDL, LDL, SBP, DBP), complications (i.e., T2DM, hypercholesteremia, hypertension diagnosis, OSA, gout, arthritis, anxiety, NAFLD, Depression), end-organ damage (i.e., Angina, myocardial infarction, heart failure, thrombosis, dyspnoea, stroke), activities of daily living and well-being. The score is used to determine an EOSS stage between 0 and 4; 0 represents no obesity-related complications, whereas 4 represents severe obesity-related disability [21, 22]. This score is prognostic, with each score correlating with an associated long-term mortality rate [23]. The Kings Obesity Staging Criteria was developed in the UK and facilitates holistic evaluation of patients living with obesity. Our team uses the modified version at Guys and St Thomas’ Hospital. It includes assessment of the Airway, BMI, Cardiovascular disease, Diabetes, Economic complication, Functional limitations, Gonadal and Reproductive axis, Health status (perceived), Body Image & eating behaviour, Oesophago-gastric junction, Kidneys and Liver. Each category is assigned a score from 0 to 3; stage 0 represents ‘normal health’, and stage 3 indicates ‘advanced disease. This criterion has considerable strength as it assesses physical health-related complications and directs HCPs to assess psychological and social well-being [24].

**Table 2:** Edmonton Obesity Staging Criteria

EOSS Stage	Definition	Complication
0	No apparent obesity-related risk factors, physical symptoms, psychopathology, functional limitations, and/or impairments of well-being	No complications
1	Presence of obesity-related subclinical risk factors, mild physical symptoms, mild psychopathology, mild functional limitations, and/or impairment of well-being	Subclinical RFs, i.e., borderling hypertension, prediabetes, NAFLD
2	Presence of established obesity-related chronic disease, moderate limitations in activities of daily living, and/or well-being	Established chronic disease, i.e., T2DM, OSAS, PCOS, hypertension, OA
3	Established end-organ damage, significant psychopathology, significant functional limitations, and/or impairment of well-being	Established end-organ damage, i.e., MI, stroke, diabetic vascular complications, heart failure
4	Severe (potentially end-stage) disabilities from obesity-related chronic diseases, disabling psychopathology, functional limitations, and/or impairment of well-being	Severe end-stage disability

Adapted from Kushner et al. [31]

#### Phenotypes of obesity based on metabolic health

Metabolic unhealthiness is often defined by the presence of abnormal parameters of blood pressure, lipids, glycaemia and systemic inflammation. Interestingly, whilst there is clear causation between obesity (BMI >  = 30) and cardiometabolic disease, research delineates a phenotype of patients with obesity who do not have metabolic complications: metabolically healthy obesity (MHO). Conversely, there are patients with normal weight who are metabolically unhealthy: ‘metabolically unhealthy normal weight’ (MUNW). These subgroups are expected, with up to 31.7% of individuals living with obesity being metabolically healthy and 23.5% of patients with normal weight being metabolically unhealthy [25]. Several epidemiological studies have demonstrated that MHO and metabolically healthy normal weight (MHNW) individuals are at a lower risk of T2DM and CVD than those who are metabolically unhealthy (MUNW, metabolically unhealthy obesity (MUO)). Epidemiological data report that MHO and MHNW have similar risks of CVD and mortality, meaning metabolic health rather than BMI may be a better surrogate of long-term risk [26, 27]. This is supported by the HUNT study, reporting that high BMI without metabolic abnormalities does not increase coronary heart disease risk [28]. Data show that fat distribution rather than BMI is more strongly associated with cardiometabolic health, underpinning the differentiation between metabolically healthy and unhealthy normal weight or obesity [27]. For instance, subcutaneous adipose tissue (SAT) is protective against cardiometabolic disease, whereas visceral adipose tissue (VAT) and ectopic fat promote cardiometabolic disease. [29, 30]. This underpins the phenotypes of MUO and MHO, whereby MHO buffer energy to the healthy SAT and MUO deposits fat in visceral and ectopic depots [8]. These phenotypes (MHO, MUNW) require further evaluation, as they can risk stratify patients to appropriate interventions, whereby MUO may need prompt intervention and MHO may not require any intervention at all. Cost-effective techniques are needed for this cohort to delineate this risk; in the clinic, this can be accomplished by assessing metabolic health via laboratory health.

#### Future obesity risk stratification research

Going forward, developing a risk stratification protocol would be helpful for obesity services, whereby an overall risk score akin to the QRISK-3 used in CVD is implemented. This score could integrate clinical findings, biochemistry, and quantitative imaging parameters. For this to occur, large-scale epidemiological studies will be required alongside robust evaluation. For patients with obesity, primary care could focus on assessing cardiometabolic complications. Screening for complications could include liver (fibrosis-4, NAFLD fibrosis risk score), cardiovascular (blood pressure, lipid profile, BNP), metabolic (HbA1c, fasting glucose), reproductive (sex-hormone binding globulin, testosterone etc.), sleep (Epworth sleepiness scale), arthritis (AIMS/AIMS2—Arthritis Impact Measurement Scales), mental health (PHQ-9) and socioeconomic complications (Simple questionnaire evaluating economic impact). [5] Perhaps digital health could aid with the implementation. Finally, the development of obesity-specific biomarkers of disease risk would be of use and may help in implementing a precision medicine approach to the management of patients living with obesity.

### Healthcare professionals approach to managing patients living with obesity

#### Communication and consultation style

Communication and consultation style in obesity management is crucial. Counselling regarding obesity in a healthcare setting is generally sub-optimal due to concerns regarding how to sensitively discuss weight, healthcare professionals (HCPs) time commitments and insufficient training at all levels [31]. Survey data indicate that patients' motivation to achieve goals is amplified when patients have positive interactions with HCPs, which may include realistic goal setting and the HCP sharing knowledge of weight loss benefits [32]. HCP should focus on the 5 A’s consultation model: Assess, advise, agree, assist, and arrange. Firstly, gaining permission to discuss weight in the clinic sensitively is essential. Open-ended questions facilitate a dialogue for shared decision-making (SDM)—SDM is particularly important as patients are more likely to adhere to interventions they actively agree to [33]. SDM aims to help patients make informed decisions, whereby clinicians provide evidence-based information while the patients present their own opinions and values. Aids like videos or leaflets can enhance decision-making. Motivational interviewing should be incorporated and is a technique which utilises goal-setting. A recent meta-analysis demonstrated more significant BMI reduction with motivational interviewing consultations, highlighting the importance of effective communication and a patient-centred approach in therapeutic decision-making [34, 35]. The language used by HCPs when assessing a patient living with obesity is vital, with research indicating that weight destigmatising language improves patient adherence to treatment. For instance, in a study which interchanged use of the terms “weight”, “BMI”, “fat” and “obesity” in obesity consultations, the term ‘fat’ was found to engender the least perceived self-efficacy in managing their condition with “obesity” being the preferred terminology for patients [36, 37]. The use of appropriate language also aids the elimination of weight stigma, which is known to affect the quality of healthcare and public health goals [38]. Overall communication and consultation style are essential aspects of the clinical evaluation of patients living with obesity. HCPs should take a holistic approach, use evidence-based consultation styles, and use terminology which is agreeable and that does not cause offence, to facilitate bidirectional patient-centred decision-making.

#### Role of primary care in the clinical evaluation of patients living with obesity

Primary care plays an essential role in initiating the clinical evaluation of patients living with obesity. Their role includes the diagnosis of obesity, clarification of metabolic risk, basic optimisation of associated complications (i.e., BP, lipids, and T2DM), intervention initiation and providing long-term continuity of care. Prompt diagnosis is crucial as this facilitates treatment and referral specialist obesity service input where appropriate. Their role can potentially reduce the prevalence of significant obesity and reduce complication severity. For instance, if weight management is instigated when a patient has prediabetes, this will help to reverse the condition and prevent diabetes development [35]. Lifestyle intervention can be delivered in primary care: including aspects of self-monitoring, goal monitoring and psychological input. When delivered in primary care, the programme should include a minimum of 12 sessions over one year, with a core session period (3 months) and a follow-up period (9 months) [33]. There is also additional scope for primary care to be involved in pharmacotherapy initiation, especially with the advent of highly effective GLP-1 therapy for obesity (i.e., semaglutide, tizepatide) [4, 39].

#### Future directions in the clinical evaluation and steps for the future

Within the UK, NHS general practice appointments are ~ 10 min, potentially limiting the ability to evaluate patients living with obesity comprehensively. Similarly, data from 18 countries, including 50% of the world’s population, indicate primary care consultations are on average 5 min or less [40]. GP appointments must be designed to accommodate extended discussions with patients, which could be facilitated by a joint-care system with the practice nurse or a primary care dietician [31]. In addition, obesity training must be prioritised as a critical theme of medical and nursing education. For instance, key concepts in obesity medicine and emerging paradigms should be integrated into medical school and the postgraduate curriculum. Training should also be on softer skills, including eliminating weight stigma, and appropriate terminology to use with patients. These initiatives may facilitate a better-equipped healthcare workforce to deal with the ever-rising obesity prevalence.

### Clinical evaluation of obesity

Clinical evaluation of patients with obesity is complex and should address physical, psychological and social well-being. It includes obesity physician evaluation via history and examination, dietitian input for dietary history and measuring body composition, laboratory investigations, evaluation of sleep oximetry and psychologist input where appropriate. Within our bariatric service in the United Kingdom (UK), we take a holistic approach. Initially, the patient has a detailed consultation with a specialist dietitian regarding their dietary history, previous dieting attempts and weight loss preferences. The patient is then assessed by an obesity physician, where a detailed history and examination are undertaken, and weight loss strategies and preferences are discussed. A team discussion is then undertaken with physicians, dieticians, psychologists, and psychiatrists present, allowing for a collaborative approach to determining the appropriate management plan. Following this, a specialist bariatric multi-disciplinary team (MDT) meeting is held to discuss each patient’s case: this is attended by an obesity physician, specialist obesity dietitian, bariatric psychologists, bariatric surgeons, sleep physicians and hepatology doctors. Here a comprehensive plan from various HCPs is devised, with onward referral to bariatric surgeons and risk optimisation.

#### Medical history

History taking is the most important part of clinical evaluation. It should focus on weight history, previous weight loss attempts, past medical history (i.e., metabolic, functional and psychosocial complications), lifestyle, family history and psychological health. The timeline of weight change is important, with coinciding life events providing aetiological clues. (i.e., new job, pregnancy, trauma/bereavement) [31, 41]. The history is also essential in determining treatment; for instance, in patients with extensive knowledge of lifestyle intervention, obesity pharmacotherapy or bariatric surgery may be more appropriate [31]. Clinicians should also take note of the degree of weight loss required to see improvement in different comorbidities. For instance 2.5 to 15% weight reduction is necessary for glycaemic improvement in T2DM, whereas 5–10% weight loss may be needed to prevent knee pain in patients with obesity [42]. These factors should be discussed with patients in deciding the optimal treatment.

#### Cardiovascular risk

Obesity is a crucial determinant of CVD risk, including hypertension, atherosclerotic disease and ischaemic heart disease. In terms of blood pressure, meta-analysis (*n* = 35 RCTs) indicates that for every 1 kg weight loss, there is a ~ 1.05 mmHg and ~ 0.92 mmHg reduction in systolic BP and diastolic BP, respectively [43]. In addition, the TOHP-II trial (lifestyle intervention in BP control) reported that individuals who reduced weight by ~ 4.5 kg in 6 months and maintained this for 3–4 years had a 65% reduced risk of hypertension [44]. In the clinic, CVD risk should be triaged by assessing for cardiac disease symptoms, history of cardiovascular disease (i.e., hypertension, dyslipidaemia, stroke, IHD) and examination. Laboratory tests should evaluate for dyslipidaemia (lipid profile), renal function (renal dysfunction increases CVD risk) and, if appropriate, BNP (Heart failure risk). It should be noted that BNP is lower in patients living with obesity; in patients presenting with acute heart failure, the mean BNP is 643 pg/ml in individuals with normal weight, 462 pg/ml in individuals living with overweight/obesity and 247 pg/ml in individuals living with severe obesity [45]. Further investigations to consider if clinical suspicion of CVD includes a transthoracic echocardiogram and ECG. If BMI is significantly raised, then echocardiogram may not be feasible due to technical considerations relating to transmission of ultrasound through adipose tissue, therefore, a nuclear medicine myocardial perfusion scan should be considered. A validated CVD risk score, such as Q-RISK 3 score, should also be calculated to define those of potential high CVD risk, guiding urgency of intervention [5]. In the evaluation, attention should also be paid to medications; Anti-obesity medications and medications used for diabetes commonly have beneficial CV effects, these medications require titration/optimisation in the context of obesity. For instance, SGLT-2 inhibitors and GLP-1 receptor agonists consistently show improvements in cardiovascular health, whereas DDP-4 inhibitors show the potential to increase heart failure hospitalisation. Therefore, if appropriate medications should be reviewed and optimised in lieu of CVD risk prevention and blood pressure optimised to appropriate targets [46].

#### Metabolic risk

BMI is a significant risk factor for diabetes and related metabolic conditions. Epidemiological data (> 100,000 nurses) identified BMI as a major risk factor for diabetes, with every 1 kg lower weight reducing diabetes risk by 16% [9, 47]. In addition, the USPSTF demonstrated that intensive lifestyle intervention in adults living with obesity and with elevated blood glucose levels can significantly benefits weight status and reduces T2DM risk [48]. Clinical evaluation of metabolic risk should include clarification of diabetes history, family history of diabetes and questions regarding diabetes symptoms. In addition, it is important to determine contraindications to GLP-1 agonists, as medications like liraglutide 3.0 mg and semaglutide 2.4 mg are now used in treating obesity. Following this examination, should evaluate for signs of insulin resistance (i.e., acanthosis nigricans), measures of central obesity (WC / WHR) and signs of Cushing's syndrome (with the most specific clinical signs being proximal muscle wasting, broad abdominal striae and thinning of skin). Biochemical investigations should include an assessment of HbA1c (T2DM/prediabetes), fasting glucose, lipid profile (metabolic syndrome) and thyroid function tests (exclude hypothyroidism). Diabetes Mellitus is diagnosed based on a HbA1c ≥ 48 mmol/mol, a fasting plasma glucose ≥ 7.0 mmol/L or a 2-h plasma glucose ≥ 11.1 mmol/L [49]. If there is clinical suspicion of type 1 diabetes via history, then antibody tests should also be considered. If evaluation raises suspicion of Cushing’s, then an overnight dexamethasone suppression test should be ordered. Management of T2DM should focus on optimising diabetes medications which promote weight loss, including GLP-1 agonists (Ozempic 1 mg – semaglutide), SGLT-2 inhibitors and metformin, though glycaemia should not be unduly compromised in attempting to avoid drugs associated with weight gain, including insulin.

#### Reproductive health

Obesity can affect both male and female reproductive health. In women, overweight, obesity and central obesity increase PCOS risk. Data from our group have demonstrated that women with obesity have a fourfold increased risk of PCOS compared to women without obesity. In addition, mendelian randomisation data report that genetically proxied BMI and adiposity increase PCOS risk [50]. In the clinic, patients should be evaluated for signs of clinical hyperandrogenism (hirsutism, alopecia, acne) and oligo/anovulation. If concerns are raised, further tests should be considered, including hormone levels (i.e., testosterone, LH/FSH, SHBG, prolactin, 17-hydroxyprogesterone, dehydroepiandrosterone sulphate) and a transvaginal ultrasound scan (to detect polycystic ovaries) [51]. In addition, fertility should be discussed for females of reproductive age. This is because BMI > 35 is an exclusion criteria to invitro fertilisation in most services, meaning in patients of reproductive age who are experiencing infertility and are living with obesity, interventions should be expedited. Patients should be aware, however, they should not become pregnant and should use appropriate contraception due to the risk of nutritional/mineral deficiency for 18 months following bariatric surgery [5]. GLP-1 agonists are contraindicated in pregnancy due to preclinical teratogenicity data in murine models.

In males, obesity can lead to hypogonadism via negative feedback loops inhibiting testosterone; for instance, 60% of male bariatric surgery candidates are living with hypogonadism [52]. Obesity is associated with low levels of SHBG, resulting in low total testosterone levels. Formulas adjusting for SHBG should be used to obtain an estimate of free testosterone. Common signs and symptoms to assess include reduced libido, erectile dysfunction, infertility and gynaecomastia. Testing for male hypogonadism should consider, if appropriate, a hormonal profile looking at early morning SHBG, LH/FSH and testosterone. If fertility is desired and hypogonadism is evidenced, then consider semen analysis and referral to a fertility clinic [5].

#### Gastrointestinal & hepatology

Obesity can contribute to significant gastrointestinal and hepatological manifestations, with associated manifestations including cholesterol gallstone disease, GORD, acute pancreatitis and non-alcoholic fatty liver disease (NAFLD) [53]. For NAFLD, there is a clear dose–response relationship, with every one unit increase in waist circumference and BMI corresponding to a 1.07 and 1.25 increased Odds of NAFLD, respectively [54]. Meta-analysis also depicts that in patients with obesity, NAFLD prevalence is 75.27%; 43.05% have non-alcoholic fatty liver (NAFL), 33.67% have non-alcoholic steatohepatitis (NASH), and 21.60% have clinically significant liver fibrosis (stages F2-4) [55]. In the clinic, important factors in the history include alcohol intake, reflux symptoms and any right upper quadrant abdominal pain (gallbladder/liver disease). The abdomen should be examined to determine the presence of hepatology and right upper quadrant symptoms. It is important to note if an abdominal wall hernia is present, as this should be highlighted for surgical repair if bariatric surgery is to be pursued. Blood tests should include liver function (ALT, AST, GGT, ALP, bilirubin) and full blood count. The Fibrosis-4 score (FIB-4) (age, platelets, ALT, AST), an index of liver fibrosis risk, should be calculated, and if raised, a fibroscan should be organized [56]. Alternatively, the enhanced liver fibrosis (ELF) score can be used if available, allowing risk determination without needing FIB-4 and fibroscan. In addition, an endoscopy should be considered if significant upper gastrointestinal symptoms are present. This is particularly important in planning bariatric surgery [5].

#### Respiratory system

In patients living with obesity, the main obesity-related respiratory conditions are OSA and obesity hypoventilation syndrome. OSA is characterised by sleep-disordered breathing and excessive daytime sleepiness resulting in reduced quality of life (QoL). OSA is associated with cardiovascular disease and cardiometabolic complications (including NAFLD and T2DM) [57]. OSA is responsive to lifestyle intervention; in the Sleep AHEAD study of patients with T2DM, intensive lifestyle intervention reduced AHI by 9.7 compared to the control arm, with > 3 × as many participants experiencing OSAS remission at one year [58, 59]. Meta-analysis has demonstrated that for every 1 unit decrease in BMI, the AHI reduces by 2.83/hr [60]. In the clinic, patients should be evaluated for dyspnoea, OSA-related symptoms (i.e., snoring, daytime somnolence) and respiratory system examination. An OSA screening tool (i.e., STOP-BANG questionnaire) can be used to determine the risk of sleep apnoea. Further tests include overnight oximetry (i.e., if high risk for OSA on screening / considering bariatric surgery), arterial blood gas (if OHS) or spirometry. It is also important to initiate prompt OSA treatment, i.e., CPAP, if OSAS is diagnosed, as this can improve overall health and reduce the risk of bariatric surgery anaesthetic complications [5].

#### Musculoskeletal system

Obesity is a significant risk factor for musculoskeletal conditions, including osteoarthritis (OA) and gout [61]. Meta-analysis reports that for every 5-unit increase in BMI, the risk of knee osteoarthritis increases by 35%. [62]. In addition, a recent cross-sectional study has reported a dose–response relationship between BMI and consequences of knee OA, with individuals in the highest BMI category experiencing increased levels of anxiety, depression, pain and physical disability [63]. Clinical evaluation should involve the assessment of any functional limitations, and symptoms of arthritis and determining whether the patient's BMI is a barrier to orthopaedic treatment. This is important in orthopaedics as they will not proceed to knee replacement surgery in certain cases until BMI is optimised. This could be used to prioritise patients to treatment [5].

#### Age & sarcopenia

As defined by Cruz-Jentoft et al. “Sarcopenia is a progressive and generalised skeletal muscle disorder involving the accelerated loss of muscle mass and function that is associated with increased adverse outcomes including falls, functional decline, frailty, and mortality” [64]. Older age can predispose to sarcopenia development, complicating BMI’s utility in this population (Table 3). As individuals age, muscle mass tends to reduce and pathogenic visceral adiposity increases; this represents a different phenotype to younger aged individuals as BMI will underrepresent adiposity. Over time a static BMI will conceal sarcopenic obesity, with evidence suggesting that alternative measurements like waist circumference (central obesity) and mid-arm muscle circumference (a measure of muscle mass) are of greater utility as they predict better mortality in this population compared to BMI [11, 65–68]. Clinicians should take time to rule out sarcopenic obesity (SO) in older individuals or those with symptoms of sarcopenia. As highlighted in the Dutch population, the prevalence of SO is 0.9–1.4%, and sarcopenic overweight (SOW) is 6.5–6.0%. In addition, the risk of SO and SOW is significantly greater in those with > 3 comorbidities, with lower levels of PA and dietary intake, which occur with ageing associated with higher SO + SOW levels [69]. In terms of diagnosis, no fixed international guidelines for the diagnosis of sarcopenic obesity exist, however, the European association for the study of obesity (EASO) consensus statements recommend screening based on clinical symptoms, questionnaires (SARC-F) and clinical suspicion (Table 4). SARC-F is a short 5-point questionnaire consisting of determination of strength, assistance in walking, rising from a chair, stair climbing and falls risk. A score of > = 4 requires further evaluation. Whilst the test has good reliability, it had low–moderate sensitivity to diagnose sarcopenia in older people, and there are limited data evaluating it in patients living with obesity [70]. Therefore, the SARC-F should be used alongside presentation and clinical symptoms to determine whether diagnostic tests are required. Based on EASO guidelines, skeletal muscle functional parameters should be considered, i.e., Hand-grip strength (HGS) or chair stand test. If the following parameters suggest SO, then body composition should be determined via DEXA or BIA. A raised fat mass and reduced muscle mass via BIA/DEXA will confirm the diagnosis. Following this SO should be staged into Stage I (no complications) and Stage II (presence of at least one complication related to SO) (Table 4) [71]. For treatment, caution and an MDT approach should be used due to the risks of interventions leading to further reductions in muscle mass. Modest calorie intake reductions (500–750 kcal/day) should be recommended, with physical activity prioritised to preserve muscle mass. The Very low-calorie diet must be avoided as this may precipitate sarcopenia [72]. Overall, in older individuals, loss of fat-free mass and abdominal adiposity are more critical than BMI when assessing the risk of obesity-related complications [65]. Further data are required to determine whether bariatric surgery or pharmacotherapy is preferable in patients living with sarcopenic obesity.

**Table 3:**
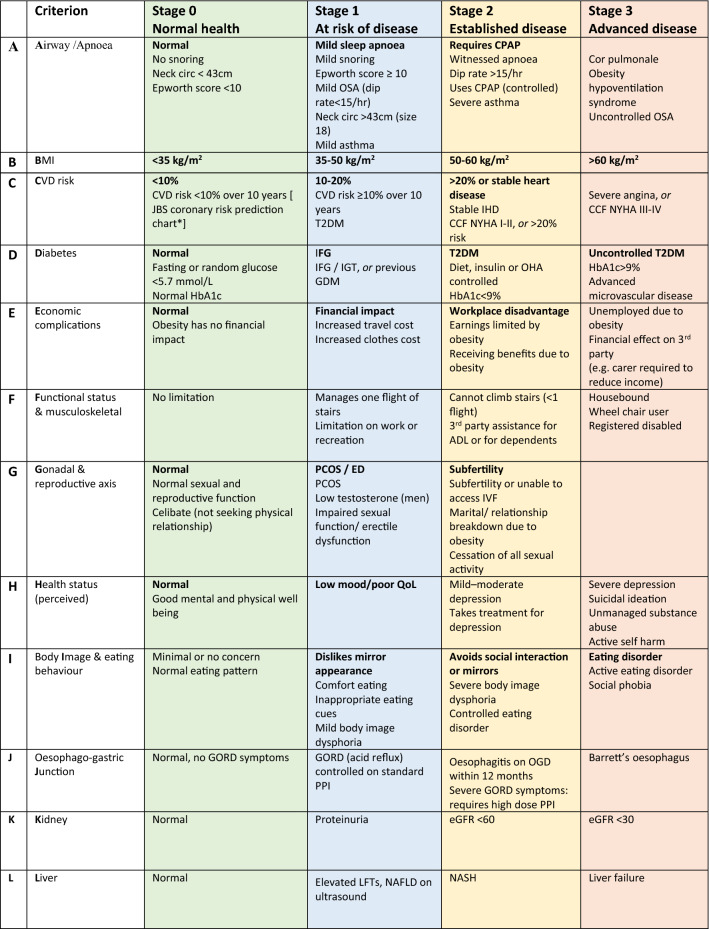
King’s obesity staging criteria Adapted from Whyte et al. [78]

**Stage 1** conditions: patients at risk of developing co-morbidities or with mild co-morbidities that may potentially justify bariatric surgery but do not need specialist opinion.

**Stage 2** conditions: established disease and higher surgical risk. This may require medical review.

**Stage 3** conditions: indicate a need for specialist opinion and may represent contraindications for surgery.

*BMI* Body Mass Index, *Neck circ* neck circumference, *CPAP* Continuous Positive Airway Pressure, *OSA* Obstructive Sleep Apnoea, *CVD* Cardiovascular Disease, *IFG* Impaired Fasting Glucose, *IGT* Impaired Glucose Tolerance, *GDM* Gestational Diabetes Mellitus, *T2DM* Type 2 Diabetes Mellitus, *GORD* Gastro-oesophageal Reflux Disease, *QoL* Quality of Life, *NAFLD* Non-alcoholic Fatty Liver Disease, *NASH* Non-alcoholic Steatohepatitis, *PPI* Proton Pump Inhibitor, *LFT* Liver Function Tests

**Table 4:** Diagnostic process for sarcopenic obesity based on ESPEN and EASO consensus statement Adapted from Fig. 1 by Donini et al. Diagnostic procedure for the assessment of sarcopenic obesity.

1: Screening	1. High BMI or WC (ethnicity-based cut-offs) 2. Surrogate markers of sarcopenia, i.e., questionnaires (SARC-F), clinical symptoms, clinical suspicion If 1 + 2 present, proceed with diagnostic process
2: Diagnosis	Two steps 1. Altered skeletal muscle functional parameters assessed via strength (i.e., handgrip strength, chair stand test) If muscle function suggests SO, proceed to body composition evaluation 2. Altered body composition: DEXA/BIA assessment showing increased FM and reduced muscle mass. (DEXA = ALM/W, BIA = SMM/W) Sarcopenic Obesity = altered body composition + altered skeletal muscle strength
3: Staging	Two-stage staging, based on presence of complications Stage I: no complications Stage II: at least one complication attributable to SO (i.e., metabolic disease, functional disability, cardiovascular disease, respiratory disease)

ALM/W, appendicular lean mass adjusted to body weight; ASMM, absolute skeletal muscle mass; BIA, bioelectrical impedance analysis; BMI, body mass index; DXA, dual X-ray absorptiometry; FM, fat mass; HGS, handgrip strength; SMM/W, total skeletal muscle mass adjusted by weight; SO, sarcopenic obesity; WC, waist circumference; SARC-F, strength, assistance with walking, rising from a chair, climbing stairs and falls [71]

**Fig. 1:**
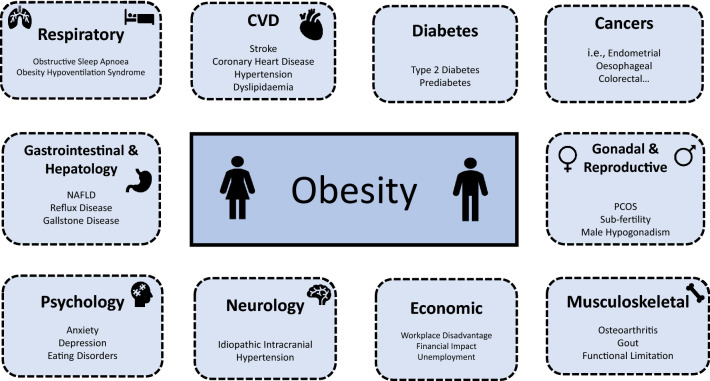
Impact of Obesity on Health. *NAFLD* non-alcoholic fatty liver disease, *PCOS* polycystic ovary syndrome, *CVD* cardiovascular disease

#### Neoplastic disease

Obesity is an established risk factor for multiple malignancies, including of the oesophagus, pancreas, colorectum, breast, endometrium and kidneys. Attention should be paid to this neoplastic risk in patients presenting to the bariatric clinic. Complexity can arise due to patients often reporting significant recent weight loss via lifestyle attempts. However, weight loss that is excessive or not warranted in the context should raise suspicion of sinister pathology. Clinical history should focus, if indicated, on enquiring regarding dysphagia, change of bowel habits and any intermenstrual/postmenopausal PV bleeding. If indicated, consider abdominal examination for abdominal masses and breast examination in situations where the patient hasn’t attended recent screening tests. Urinalysis, which should be performed on all patients presenting to the obesity clinic, should be assessed for any haematuria [5, 73].

#### Neurology

Obesity is linked to the syndrome of idiopathic intracranial hypertension (IIH). This commonly presents with high-pressure headache (worsened by lying down/Valsalva manoeuvre and in the morning) with associated visual symptoms (i.e., blurred vision, diplopia). If history is of concern, examination findings for IIH include papilloedema on fundoscopy, reduced visual acuity or in advanced cases, VIth nerve palsy. Abnormal findings necessitate urgent referral to ophthalmology and neurology for assessment. Diagnosis is reached by lumbar puncture, showing opening pressure > 25 cm H2O with cerebrospinal fluid having normal composition [5]. IIH is responsive to weight loss with bariatric surgery, significantly reducing intra-cranial pressure. For instance, data show that 24% body weight reduction can lead to IIH remission, with roux-en-y gastric bypass (RYGB) being the superior surgical procedure. Therefore, surgical approaches should be highlighted to patients presenting with IIH [74, 75]. Furthermore, in a recent RCT of patients with BMI ≥35, women with idiopathic intracranial hypertension who underwent bariatric surgery experienced disease remission and reduced ICP [5, 75].

#### Psychiatric

As highlighted via systematic review, there is a bidirectional relationship between obesity and depression. Mechanisms underpinning this relationship: (1) obesity is an inflammatory process, with inflammation associated with depression, (2) Obesity contributes to HPA axis dysregulation, which could contribute towards depression, (3) Obesity can reduce self-esteem, a risk factor for depression. Overall the risk of depression in obesity is increased by 55%, whereas depression increases the risk of obesity by 58%. This shows that an assessment of mood should be performed in all patients living with obesity [76]. In addition, it is also important to assess for the presence of eating disorders, particularly binge-eating disorder (BED) and bulimia nervosa (BN). The key diagnostic criteria for both BED and BN are recurrent eating of excessive amounts of food with sense of lack of control during the episode. Questionnaire-based screening tools are appropriate in the first instance, as patients often find it challenging to discuss eating disorders in a consultation. When patients are highlighted as having potential eating disorder, psychological interventions should be offered [5].

## Conclusion

Overall, the clinical evaluation of the patient living with obesity is important; it should take a holistic approach and involve obesity physicians, dietitians, psychologists and other healthcare professionals. Whilst BMI is used for diagnosis, it is limited, and risk stratification tools like Kings Obesity Staging Criteria should be used clinically. During the evaluation, a detailed history should be taken alongside examination, laboratory tests and further investigations when warranted. Future research should focus on delineating optimal risk stratification tools to delineate the appropriate therapy in patients living with obesity.

## Data Availability

Not applicable, this is a review article.
